# Supplementary material for: Antibody responses to Sinopharm/BBIBP-CorV in pregnant mothers in Sri Lanka
Source: PLOS Glob Public Health. 2022 Jul 18;2(7):e0000607. doi: 10.1371/journal.pgph.0000607 (PMC10022122; doi:10.1371/journal.pgph.0000607)
Supplement: Attachment — Submitted filename: Answers to reviewer questions 11.05.2022.docx [file pgph.0000607.s002.docx]

11^th^ May 2022

Abram Luther Wagner, PhD, MPH

Academic Editor

PLOS Global Public Health

Dear Professor Wagner,

**Submission of manuscript ‘Antibody responses to Sinopharm/BBIBP-CorV in pregnant individuals in Sri Lanka’**

We wish to thank the reviewers for carefully going through our manuscript and the useful suggestions they have made. We have incorporated all these changes in the revised version and letter and have addressed all issues raised by the reviewers. We have highlighted the changes in the revised manuscript.

Reviewer #1: This study explores a timely topic and can provide relevant information on COVID-19 response.
**Data availability: PLOS journals require authors to make all data necessary to replicate their study’s findings publicly available without restriction at the time of publication. When specific legal or ethical restrictions prohibit public sharing of a data set, authors must indicate how others may obtain access to the data.**

Response: We thank the reviewer for this important suggestion. We agree that sharing data and stating how data can be obtained for independent analysis is very important. We have mentioned that the primary data used for the analysis (excel sheets) can be requested by the corresponding author of the manuscript.

**Regarding sampling, what was the inclusion and exclusion criteria for participants and justifications (if any)? Why is the sample size low?**

Response: We thank the reviewers for this very important question. Our inclusion criteria for this paper were females 18 years and above, who were registered with the maternal and child health clinics in the Colombo Municipal Council region of Sri Lanka. All those who received the first and second doses of the Sinopharm/BBIBP-CorV vaccine and who we were able to give a follow up blood sample, 6 – 12 weeks after the second dose were included in the study. Pregnant females below the age of 18 were excluded from the study, as consent would be an issue in such patients as they are not legal adults. The sample size is low due to poor patient turn up for the follow up blood sample, as during that time Sri Lanka was going through another wave of COVID and patients were unable to travel to the centers to give their follow up samples.

**Was the distribution of participants among the 3 trimesters by design or co-incidental, how did this affect the study outcome?**

Response: We thank the reviewer for this important question. The distribution of the participants among the 3 trimesters was not by design and we recruited all pregnant mothers who received both doses of the vaccines and were available to give a follow up blood sample 6 to 12 weeks later. We believe that the distribution of the participants among the three trimesters was unlikely to have affected the study outcome, as patients from all trimesters had similar seroconversion rates and there were no reported maternal or fetal adverse events.

**Reviewer #2: The authors use “Pregnant individuals”, “Pregnant Women”, and “pregnant females” should be consistent with the population. I would suggest they use “Pregnant mothers”**

Response: Thank you for this suggestion to use this terminology. We have gone through the manuscript and edited it as advised.

**Materials and methods
Start line 107 with a word not a number (i.e. Ninety-four (94) females above….) and line 167**

Response: We thank the reviewer for bringing this to our attention. We have made the corrections and highlighted it in the revised manuscript.

**Was it part of the inclusion criteria for all the participants to be above 18 years? Or it was 18 years and above. Clarify on this.**

Response: Yes, it was a part of our inclusion criteria for all the participants to be 18 years of age and above. We only obtained ethics approval to include adult individuals in our study and therefore, we did not recruit any pregnant mothers below the age of 18.

**“These individuals received the first dose of the vaccine between mid July 2021 to
110 mid-August 2021” Were all the participants in the same trimester of their pregnancy? Add more information on this.**

Response: Thank you for this important question. All participants were not in the same trimester of their pregnancy. As we have mentioned samples were collected from all patients who were 18 years and above who came to the clinic to receive their vaccination. As most often patients were above 12 weeks in their pregnancy, most individuals who were recruited to the study were either their second or third trimester. As mentioned in the results, only 2/94 (2.1%) women obtained the vaccine in the first trimester, 57/94 (60.6%) in the second trimester and 33 (35.1%) in the third trimester

Thank you for considering our manuscript for publication with PLOS Global Public Health.

Yours Sincerely,

Prof. Neelika Malavige
